# In vitro anti-cancer activity of doxorubicin against human RNA helicase, DDX3

**DOI:** 10.6026/97320630012347

**Published:** 2016-10-18

**Authors:** Mahendran Botlagunta, Bhulakshmi Kollapalli, Lavanya Kakarla, Siva Priya Gajarla, Sai Pujitha Gade, Chandra Lekha Dadi, Akhila Penumadu, Shaik Javeed

**Affiliations:** 1 Department of Biotechnology, K L University, Guntur, Andhra Pradesh-522502, India; 2 Sweety Biologicals India Private Limited,Kavali, Andhra Pradesh-524201, India

**Keywords:** anti-cancer activity, doxorubicin, RNA helicase, DDX3

## Abstract

RNA helicase, DDX3 is a multifunctional enzyme and is known to be associated with several diseases like HIV progression, brain and breast cancer. Some of the ring expanded nucleoside compounds such as REN: NZ51, fused di imidazodiazepine ring (RK33), (Z)-3-(5- (3-bromo benzylidene)-4-oxo-2-thioxothiazolidin-3-yl)-N-(2- hydroxy phenyl) propanamide compound (FE15) have been documented to inhibit DDX3 helicase activity. However, synthesis of these drugs is limited to few research groups. Prevalence of literature study, we found that doxorubicin form strong hydrogen bond interactions with crystallized form of DDX3 using in-silico molecular docking approach. To evaluate the biological inhibitory action of doxorubicin, we performed the ATPase activity assay and anti-cancer activity using H357 cancer cell lines. Results showed that doxorubicin continually declined the inorganic phosphate (Pi) release and inhibited the ATP hydrolysis by directly interacting with DDX3. Anticancer activity was detected by MTT assay. The half maximal inhibitory concentrations of doxorubicin (IC50) for H357 cancer cell line is 50 μM and also doxorubicin significantly down regulated the expression of DDX3. Taken together, our results demonstrate, that inhibition of DDX3 expression by using doxorubicin can be used as an ideal drug candidate to treat DDX3 associated cancer disorder by interacting with unique amino acid residues (Thr 198) and common amino acid residues (Tyr 200 and Thr 201).

## Background

RNA helicases mainly found in all eukaryotes and most prokaryotes. These were distinguished from others based on highly conserved four amino-acid residues (Asp (D)-Glu (E)-Ala (A)-Asp/His (D/H)) at N-terminal region and helicase domain at C-terminal end [1,2]. This amino acid motif has been found in more than 500 proteins and has shown to represent up to 2% of the open reading frames of a genome and may share overlapping or distinct biological cellular functions [3]. These proteins have shown to associate with several aspects of energydependent RNA metabolism including translation, ribosome biogenesis, and pre-mRNA splicing [4,5]. Unlike DEAH box genes, DEAD box genes have been identified on all human chromosomes except 15, 18, and 21. This highlights the importance that DEAD box RNA helicases in the physiological management of the cell and its survival. Among several DEAD box RNA helicases, DDX3 is located on X-chromosome at Xp11.3-p11.23 [6]. It has a functional homologue on the Y chromosome, DBY, and this gene product has an activity that is crucial for normal spermatogenesis [7,8,9]. It has shown to participate in human folliculogenesis and its deletion has shown to represent an important genetic cause of primary amenorrhea or impairment of female fertility [10].

Human DDX3 encodes a transcript of 5.3 kb in size that encodes a polypeptide of 662 amino acids and this protein has 9 conserved domains and every domain has shown to play very important role in several aspects of RNA metabolism (Table 1). DDX3 was crystallized with the help of Adenosine Mononucleotide (AMP), this crystallized DDX3 has two distinguishable domains comprised of N-terminal DEAD box domain 1 (211-403 residues) and C-terminal helicase domain 2 (411-575 residues). Both domains displayed Rec A-like folds comprising a central β-sheet flanked by α-helices connected by a non-canonical linker of 11 amino acids [11]. Moreover, elevated expression of DDX3 were found to be greatly in a highly aggressive metastatic breast cancer cell line, MDA-MB-231, as compared with non-metastatic MCF-7 cells, which indicates its potential role in aggressive breast cancers and the associated metastatic diseases [12,13]. We have previously demonstrated that over-expression of DDX3 in immortalized nonturmorogenic MCF10A cells promoted neoplastic transformation as indicated by the down regulation of E-cadherin. It is a common feature of a variety of metastatic epithelial tumors including those of lung, breast and prostate cancer [14,15,16]. Hypoxic regions of solid tumors were considered to be the primary sites for the generation of the metastatic phenotype and have been demonstrated to be chemo and radio-resistant [17, 18,19,20,21]. We have demonstrated that hypoxia inducible factor HIF-1 induced the expression of DDX3 in two different breast cell lines by binding directly or indirectly to the hypoxia-response element (HRE) in the DDX3 proximal promoter [22]. On the other hand a significant down regulation of DDX3 expression is found in hepatocellular carcinoma (HCCs) from hepatitis B virus (HBV)-positive patients, but not from HCV-positive ones, compared to the corresponding non tumor tissues [23]. In hepatocellular carcinoma model DDX3 was found to act as tumor suppressor by activating the expression of cyclin dependent kinase inhibitor p21cip1 [13]. Besides the cancer, induced expression of DDX3 also found in HIV-1 infected cells [24,25]. Overall it suggests that DDX3 is a multifunctional protein and the regulatory mechanisms and signaling pathways of DDX3 is disease specific.

In recent days DDX3 is getting more attention, due to its association not only in embryonic development but also in multiple diseases like HIV, neuro-degenerative diseases, hepatocellular carcinoma, brain and breast cancer. Few molecules have been discovered to inhibit the function of DDX3 by blocking the function of the helicase activity [26,27, 28,29,30]. Biochemical analysis showed that, those molecules effectively inhibited the helicase enzyme activity, but no structural analysis was performed to elucidate the direct association of those molecules with DDX3. Therefore, in the present study, we made an attempt on in-silico molecular docking approach to identify the binding site interactions between doxorubicin and DDX3.

## Methodology

### Protein (receptor) preparation

Crystal structure of human DDX3 (PDB ID 2I4I) of resolution 2.20 Å with respective ligand, AMP was retrieved was retrieved from RCSB protein data bank. Hydrogen atoms were added, and then pdb file is uploaded to make receptor as per the standard method by using Fast Rigid Exhaustive Docking (FRED) [31].

### Ligand Preparation

NZ-51 was retrieved from published papers and fused diimidazodiazepine compound RK-33 retrieved from United States Patent Application Number 20110275588. The conformational space of the compounds was employed using omega (optimized ensemble generation application) program from Open Eye Scientific Software (http://www.eyesopen.com/omega). In our computations, we generated a maximum of 500 conformers per molecule as a default and build as a single database per molecule. It is due to the fact that, increasing the number of conformations can have the chances of finding better pose. Fast Rigid Exhaustive Docking [FRED 3.0.0] was used in this study to dock the OMEGA 2.4.6 pre-generated multi-conformer library mentioned above. FRED filters the poses based on enough contact with the receptor. Fred dock/score all possible positions of each ligand in the binding site and clash poses with the protein get rejected from the docking analysis. The final poses are scored using chemgauss4 score as default parameter. The filtered compounds were docked into the binding site of DDX3 (PDB code: 2I4I).

### Cell lines and culture conditions:

Human OSCC lines H357 was obtained from European Collection of Cell culture (ECACC). H357 cells were maintained with DMEM/F12 (Gibco.1133005) supplemented with 10% fetal bovine serum, 2 mM L-glutamine, 0.5 mg/ml sodium hydrocortisone succinate in a humidified atmosphere of 5% CO2 at 37°C and passaged every 1-2 days to maintain logarithmic growth.

### MTT assay:

H357 cells were trypsinized with 0.25% trypsin, 0.1% EDTA solution and the cells were counted using TC-10 automated cell counter (BioRad) and 1500 cells were plated in 96 well plates (BD Biosciences. 353072) for overnight. Next day media were changed and treated with variable concentrations of ketorolac salt (Sigma.K1136). After 48 hours the plate was treated with MTT (3- (4,5-dimethylthiazol-2-yl)-2,5-diphenyltetrazolium bromide), (Sigma.M2128) at a concentration of 0.5 mg/ml in 100μl of complete media and kept in the 370 C incubator. After 4 hours, media were completely removed and formazan crystals were dissolved in dimethyl sulphoxide (SRL.042982) and the absorbance of the colored solution was quantified at 590 nm with the help of Varioskan Flash Multimode Reader (Thermo Scientific™). The data were analyzed using MS office excel, 2010.

### ATPase activity assay:

DDX3 protein was purified from as per the standard protocol [22]. In brief, bacterial cell lysis was passing through Ni-NTA agarose resin (In vitrogen) and the protein was purified by affinity chromatographic method. Prior to performing ATPase activity, the purity of the protein was confirmed by western blotting with custom-made anti-DDX3 antibody. For ATPase activity, malachite green assay was performed to measure the production of inorganic phosphate during ATP hydrolysis by DDX3 as described by standard method [32,33].

## Results

### Identification and characterization of active site amino-acid constraints for DDX3

**Figure 1:**
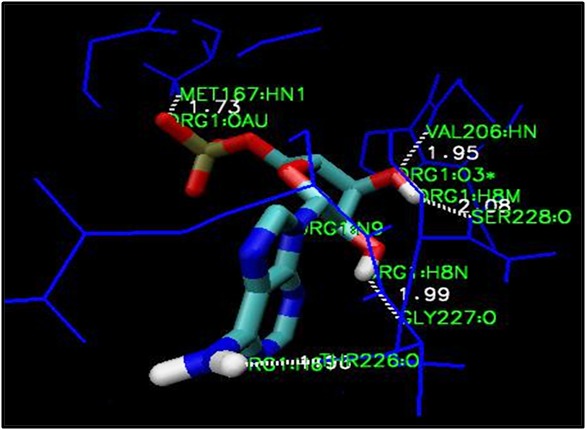
AMP docked into the ATPase binding site of DDX3. Dotted lines show the hydrogen bond interaction between ligand and active site amino acid atoms.

To identify the molecular shape and docking constraints of the ligand (AMP) we generated a box using molecular cavity detection algorithm in receptor setup workflow module at Open Eye software. The box dimensions for crystallographic AMP displayed dimensions as 27.00 Å x 22.00 Å x 26.00 Å with a box volume. Five flexible amino-acid constraints (Gly 227, Ser 228, Thr 226, Met 167 and Val 206) were detected with AMP and they possessed five hydrogen bond interactions (Figure 1).

### Receptor based molecular docking of small molecule inhibitors against DDX3

**Figure 2:**
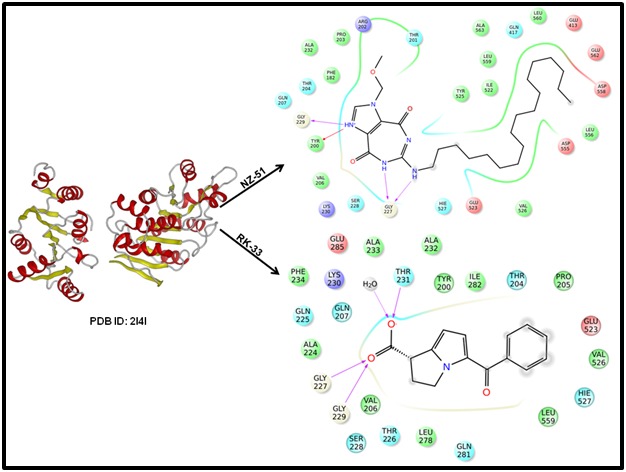
A close-up view of amino acid residues within a 5 Å distance of the binding site of A) Hydrogen bonding interactions between NZ-51 and DDX3. B) Hydrogen bonding interactions between RK33 and DDX3.

Ring-expanded nucleosides (REN) as a potential DDX3 inhibitor based on biochemical assays and named as NZ-51. The NZ51 molecule is a 4,5-Dihydro-8H-6-(N-octadecyl)amino-1-(2-deoxy-- D-erythropentofuranosy1)-imidazo[4,5-e][1,3]diazepine-4,8 dione with total nominal mass of 563 Da and with a molecular formula of C29H49N5O6. Although, this compound has shown to possess antiviral activity, no structural data is available to elicit the binding constraints. In order to understand the structural dynamics of NZ51, top 10 conformational poses were docked against DDX3 to identify the best pose fit. Among 10 poses, we have selected single conformational pose with the lowest energy for NZ-51 as -4.382 K.cal/mol. Some important interactions between NZ51 and DDX3 include H-bonding between Tyr 200- HO---HN at N1 position of NZ-51 and internal hydrogen bond between amino acid residues in presence of NZ51 are as follows Gln 523---Gln 523, Gly 229---Gln 207, Gln523…His 527, Glu 285--- -Tyr 200, Gly 229….Ala 233, Gln 207…Thr 204. Moreover, two additional hydrogen bond interactions were detected between two water residues at 670 and 692 with Glu 285 and Thr 231 respectively. Some important hydrophobic amino acid residues surrounding the long alkyl chain of NZ-51 include Ala 232, Ala 233, Pro 203, Phe 182, Lys 230 and Arg 202 (Figure 2a). On the other hand, tri cyclic 5:7:5-fused di imidazo di azepine ring (RK- 33) system containing compound were recently found to possess antitumor activity in a series of cancer cell lines possibly by regulating the expression of DDX3 [28]. However, the structural interaction of this compound with DDX3 is not elucidated till know, therefore we performed rationale molecular docking using FRED approach. RK-33 made nine hydrogen bond contacts with various amino acids with the lowest energy of -2.3 K.cal/mol. Gln-207-NH…NH is in direct association with imidazole ring of RK33 at 19th position. Six internal hydrogen bonds were detected between Gln 285---Tyr 200, Gly 229---Gln 207, Gly 229…Ala 233, Ser 228---Thr 204, Thr 204…Gln 207 and Lys 208---Thr 204. In addition, three more hydrogen bond interactions were detected in presence of water molecules between Gln285---OH-670, Thr 231---0H-692, Thr 226---OH-773. Some important hydrophobic amino acid residues around the RK33 include Phe-182, Tyr-200, Thr-201, Arg-202, Pro-203, Thr-204, Pro-205, Lys-208, Thr-226, Gly-227, Ser-228, Gly-229, Lys-230, Thr-231, Ala-233, Glu-285, Arg-503 and His-527 (Figure 2b).

### Comparative analysis of small molecule inhibitors against DDX3

**Figure 3:**
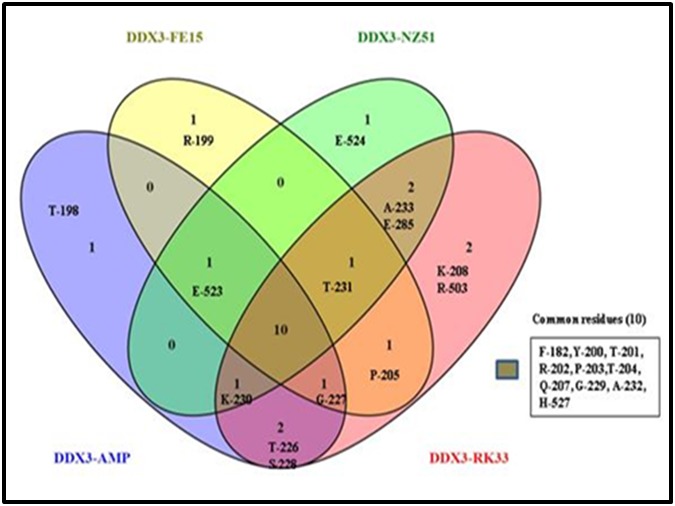
Venn diagram depicting the common and unique amino acid residues between AMP, FE15 and NZ51 and RK33.

In an effort to determine, which amino acids might be contributing to DDX3 inhibitory activity, we performed the comparative analysis between NZ-51, RK33 and FE15 (For FE15, intra and inter hydrogen bond interactive amino acid residues were derived from published article [36] using a Venn diagram (http://bioinfogp.cnb.csic.es/tools/venny/index.html) (Figure 3a). The venn diagram suggest that several hydrogen and nonbonding interactions were detected with different amino acid residues starting from Phe 182 to Glu 285 and Arg 503 to His 527 with variable ligands tested in our study. Among all RK33 displayed two unique amino acids such as Lys 208 and Arg 503. Similarly, Glu 524, Arg 199 and Thr 198 for NZ51, FE15 and AMP around the cavity (Figure 3b). On the other hand 10 amino acid residues Phe 182, Tyr 200, Thr 201, Arg 202, Pro 203, Thr 204, Gln 207, Gly 229, Ala 232 and His 527 were found to be common around the cavity of the all drugs tested including AMP.

### Doxorubicin inhibits the ATPase activity of DDX3

**Figure 4:**
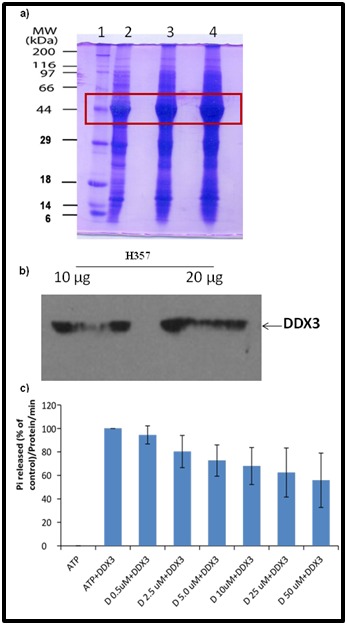
Binding assay for His-DDX3 to Doxorubicin salt. (A) SDS-PAGE and coomassie staining showing 1) uninduced and 2,3,4) induced His-DDX3. (B) Western blot was performed using polyclonal anti-DDX3 antibody. (C) Binding of Doxorubicin salt to His-DDX3.

To identify the role of doxorubicin on DDX3 ATPase activity, initially we cloned the full length human His-DDX3 protein and over expressed by bacterial system. Later the purity and identity of DDX3 protein were confirmed by SDS-PAGE (SDSpolyacrylamide Gel Electrophoresis) (Figure 4a) and by immunoblot analysis using DDX3 specific antibodies (Figure 4b). Then we incubated the purified His-DDX3 (6μM) with increasing concentrations (0.5, 2.5, 5.0, 10, 25 and 50 μM) of doxorubicin and measured the ATPase activity by malachite green assay. The result indicated that the addition of doxorubicin salt to DDX3 resulted in continual decline in the inorganic phosphate (Pi) release with respect to control untreated group (Figure 4c). However, when the DDX3 protein was incubated with 50 μM concentration, the release of pi was reduced by approximately 50%. This result suggests that doxorubicin salt inhibits the DDX3 ATPase activity in a dose dependent manner. Overall, the above data suggests that doxorubicin salt inhibit its ATPase enzyme activity.

### Doxorubicin inhibits DDX3 protein expression and reduces the cell viability in H357 cells

**Figure 5:**
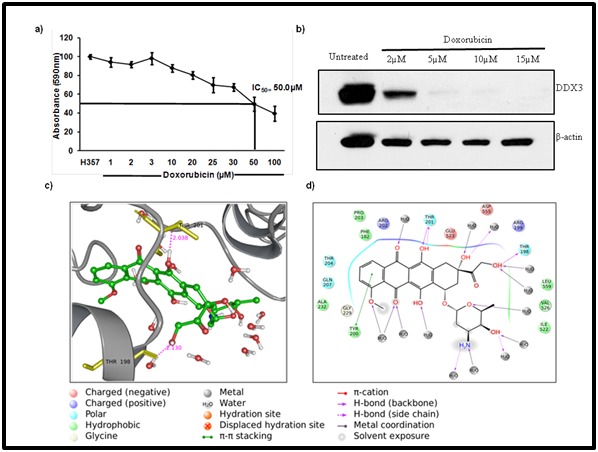
Doxorubicin salt inhibits the DDX3 protein expression and reduces the cell viability in OSCC. A) H357 cells were treated with indicated amount of Doxorubicin for 48h and cell viability was determined by MTT assay. B) Western blotting was performed to detect the expression of DDX3 and β-actin. C) The ligand interaction is depicted in the binding pocket of the target protein (2I4I) along with hydrogen and non-hydrogen bond interactions. D) Schematic drawing of types of interactions of the ligands using lig plot.

To study the anti-cancer activity of doxorubicin on OSCC cells, the H357 cells were incubated with various concentration of doxorubicin for 48hr’s and cell viability was determined by MTT assay. As shown in (Figure 5a) doxorubicin was able to decline the cell growth from 1 μM and it continues until 100 μM concentrations. The half maximal inhibitory concentration (IC50) of doxorubicin in H357 cells is 50 μM. Further, our immunoblot study suggests that doxorubicin significantly reduced DDX3 protein expression levels as compared to DMSO treated cells (Figure 5b). Later, the molecular interaction of the doxorubicin with DDX3 was confirmed by molecular docking analysis. Results showed that, doxorubicin form a strong hydrogen bond interactions with Thr 198, Thr 201 (Figure 5c) and π-π stacking with Tyr 200 amino acid residues (Figure 5d). Overall, it suggests that doxorubicin directly interacts with DDX3 by forming intra and inter molecular interaction with active site amino acid residues.

## Discussion

Ring expanded nucleoside molecules (REN), analogues has shown to inhibit the activity of viral NTPase/ helicase activity by incorporating into nucleic acids during transcription of a DNA/ RNA template by a DNA or RNA polymerases [27,34, 35,36,37]. The NZ51 is recently identified ring expanded nucleoside molecule (REN), where the six membered ring of the natural purine heterocycle has been expanded to a seven membered ring [38]. More importantly, this compound has inhibited DDX3 helicase activity in vitro and had no toxicity in mice, at concentrations that inhibited the enzyme activity [39]. Although NZ-51 inhibited DDX3 enzymatic activity direct inhibition of RNA helicases has not been proven. Therefore, we employ in silico molecular docking approach to understand the structural dynamics of NZ- 51 with DDX3 using FE15 as a reference drug. Our results showed that NZ-51 interacted with Tyr 200 rather than Gln 207 in case of FE15, it suggest both drugs may take distinct metabolic pathways to inhibit the ATPase activity of DDX3. Moreover, the interaction of NZ-51 with Tyr 200 further supports the inhibitory role of REN analogues on purine/pyramidine metabolism in cancer cells by kinase inhibitors as described earlier [40,41,42]. Apart from NZ-51, tricyclic 5:7:5-fused diimidazodiazepine ring (RK-33) compound in combination with radiation has shown to reduce the formation of colonies in lung by blocking the progression of cells from G1 to S phase [26]. However, no structural data is available to understand the molecular interaction of RK33 with DDX3. By rational molecule modeling and docking approach we found that RK33 formed a strong hydrogen bond interaction with Gln 207 with 1.97 Å distance as similar to FE15. Along the lines, we found that doxorubicin form a strong inter and intra molecular interaction with human RNA helicase, DDX3 and thereby inhibit the multiple DDX3 associated disorders. Our hypothesis is further augumented by ATPase inhibitory activity of DDX3 by doxorubicin and also the down regulation of DDX3 gene expression by increasing the concentration of the drug. On the other hand this drug formed a hydrogen bond interaction with Thr 198, one of the unique amino acid interactions between DDX3 Vs AMP and Tyr 200 and Thr 201, common amino acid residues across the all drugs tested in our study.

## Conclusion

In this study, we investigated the role of doxorubicin on DDX3 protein by in silico molecular docking studies, DDX3-ATPase activity inhibition and expression of this protein levels in H357 cancer cell lines by using MTT assay. Collectively, our results showed that doxorubicin significantly reduced ATPase activity, protein expression levels in cancer lines and also showed binding site interactions with unique amino acid residues (Thr 198) and common amino acid residues (Tyr 200 and Thr 201) in DDX3. By comparing these results we concluded that doxorubicin is an ideal drug candidate to treat cancer associated disorders.

## Competing interest

There is no competing interest.

## Figures and Tables

**Table 1:** Conserved domains of human DDX3 protein.

S. No	Name of the motif	Signature
1	Q	FTTROTOVQ
2	I	AQTGSGKT
3	Ia	PTRELA
4	Ib	TPGR
5	II	DEAD
6	III	SAT
7	IV	FVET
8	V	RGLD
9	VI	HRIGRTGR
